# Biological nanoparticles carrying the Hmda-7 gene are effective in inhibiting pancreatic cancer *in vitro* and *in vivo*

**DOI:** 10.1371/journal.pone.0185507

**Published:** 2017-10-06

**Authors:** Qingyun Zhu, Xinting Pan, Yunbo Sun, Zhengbin Wang, Fuguo Liu, Aiqin Li, Zhihui Zhao, Yunlong Wang, Kun Li, Liangyu Mi

**Affiliations:** 1 The Affiliated Hospital of Qingdao University, Qingdao, China; 2 Nano New Material Key Laboratories of Qingdao University, Qingdao, China; 3 Department of ICU, the Affiliated Hospital of Qingdao University, Qingdao, China; University of Florida, UNITED STATES

## Abstract

**Objectives:**

Pancreatic cancer is one of the most common malignancies of the digestive system, and remains a clinical challenge. This study aimed to assess the effects of bovine serum albumin (BSA) nanoparticles carrying the hMDA-7 gene (BSA-NP-hMDA-7) in the treatment of pancreatic cancer.

**Methods:**

BSA-NP-hMDA-7 was generated by nanotechnology and gene recombination technology. A total of 5 BXPC-3 or PANC-1 pancreatic cancer cell groups were examined, including Control, BSA-NPs, Empty vector, hMDA-7 plasmid, and hMDA-7 BSA-NPs groups, respectively. Proliferation and apoptosis of cultured cells were assessed by the MTT method and flow-cytometry, respectively. In addition, pancreatic cancer models were established with both cell lines in nude mice, and the expression profiles of hMDA-7 and VEGF in cancer tissues were measured by Western blot and immunohistochemistry.

**Results:**

BSA-NP-hMDA-7 nanoparticles were successfully generated, and significantly inhibited the proliferation of BXPC-3 and PANC-1 cells; in addition, apoptosis rates were higher in both cell lines after treatment with BSA-NP-hMDA-7 (P<0.05). Nude mouse xenograft studies indicated that treatment with BSA-NP-hMDA-7 nanoparticles resulted in decreased tumor size. Moreover, the hMDA-7 protein was found in tumor tissues after hMDA-7 gene transfection, while BSA-NP-hMDA-7 significantly suppressed VEGF expression in tumor tissues. Similar results were obtained for both BXPC-3 and PANC-1 xenograft models.

**Conclusion:**

BSA nanoparticles carrying the hMDA-7 gene effectively transfected BXPC-3 and PANC-1 pancreatic cancer cells, causing reduced cell proliferation and enhanced apoptosis in vitro. In mouse xenografts, BSA-NP-hMDA-7 treatment decreased tumor size and reduced VEGF expression. These findings indicated that BSA-NP-hMDA-7 might exert anticancer effects via VEGF suppression.

## Introduction

Pancreatic cancer is one of the most common malignancies of the digestive system [1–3]. With 337,872 new cases occurring annually, pancreatic cancer is the 12^th^ common cancer [4] and the seventh leading cause of cancer related mortality with 331000 deaths per year [5]. It is characterized by high malignancy, rapid progression, vascular invasion, neurotropic growth, and unfavorable patient outcome, with median survival of 3–6 months and a 5-year survival rate of less than 5% [6]. Combinational chemotherapies are used for pancreatic cancer treatment, e.g. FOLFIRINOX (fluorouracil [5-FU], leucovorin, irinotecan and oxaliplatin) and gemcitabine/nab-paclitaxel; however, gemcitabine remains the standard of care for pancreatic cancer therapy [7]. Despite the available treatment options, pancreatic cancer incidence rates are almost equal to mortality rates [8]. Therefore, there is an urgent need for new and effective therapeutics for this deadly disease.

Interestingly, gene therapy is considered a very promising tool for treating pancreatic ductal adenocarcinoma [9]. High efficiency with sustained release is critical for gene therapy against tumors. Indeed, biological nanoparticles cross-linked with plasmid DNA could penetrate blood pancreatic barrier and blood brain barrier; they also avoid interception by the reticuloendothelial system while maintaining sustained release, and are therefore superior to viral carriers regarding the immunogenicity and potential effects on tumorigenesis [10–12]. Albumins, a group of proteins with small molecular weights, are abundant in the serum and can be used as biological nanocarriers, with the advantages of large surface, no toxicity, biodegradability, and high efficiency of DNA transfection into cancer cells, which make albumin nanoparticles an ideal gene delivery system [13].

Melanoma differentiation associated gene (mda-7) is a tumor suppressor gene, whose transfer suppresses cell growth and enhances apoptosis in various cancers via multiple intracellular signaling pathways [14]. We hypothesized that bovine serum albumin (BSA) nanoparticles carrying the hMDA-7 gene will be effective in the treatment of pancreatic cancer. Therefore, this study aimed to assess the effects of BSA nanoparticles harboring hMDA-7 (BSA-NP-hMDA-7) on pancreatic cancer cells.

Interestingly, BSA-NP-hMDA-7 were effectively transfected into BXPC-3 and PANC-1 pancreatic cancer cells, respectively, which resulted in decreased cell proliferation and increased apoptosis *in vitro*. *In vivo*, BSA-NP-hMDA-7 treatment decreased tumor size and VEGF expression in mouse pancreatic tissues. These findings indicated that BSA-NP-hMDA-7 might be used for the treatment of pancreatic cancer.

## Material and methods

### Cell culture

The human pancreatic cancer cell line BXPC-3 was from cell bank of Chinese Academy of Sciences (Shanghai, China). PANC-1 cells were purchased from Shanghai Kang Lang biological technology co., LTD. BXPC-3 and PANC-1 cells were cultured in RPMI-1640 (Sigma-Aldrich, USA) supplemented with 10% FBS and 1x antibiotic/antimycotic solution (Sigma-Aldrich) in a humidified atmosphere containing 5% CO_2_ at 37°C.

### BSA-NP-hMDA-7 preparation

The pcDNA3.1-hMDA-7 plasmid (a generous gift from Professor Jicheng Yang of the Department of Molecular Biology, Suzhou University) was constructed by inserting the hMDA-7 DNA fragment from the Puc19- hMDA-7 plasmid into pcDNA3.1 through Kpn I and Xba I restriction sites. The recombinant plasmid was transformed into DH5α competent cells by the CaCl_2_ method.

A total of 0.1 mg pcDNA3.1-hMDA-7 plasmid DNA dissolved in Tris-EDTA buffer was mixed with 3 ml of 2% BSA (Sijiqing Company, China), and water was added to a total mixture volume of 5 mL. Absolute ethyl alcohol (6.5 mL) was gradually added as well as 40% glutaraldehyde, and incubated at room temperature overnight. After centrifugation, the supernatant was discarded and the precipitate dissolved in water after ethanol evaporation. The solution was submitted to high speed centrifugation on an ultracentrifuge from BECKMAN (USA), and BSA nanoparticles obtained in the supernatant were stored at -20°C. Blank BSA nanoparticles (BSA-NP) were prepared as described above but without the hMDA-7 plasmid DNA. BSA-NP morphology and size were examined on a JEM-1200EX transmission electron microscope (HITACH, Japan). Zeta-potential was measured on a Zetasizer 3000HS Particle size analyzer (Malvern Instruments, UK).

### Cell grouping

BXPC-3 and PANC-1 cells were randomly classified into five groups and seeded into 96-well plates at 2×10^5^ cells/well (six replicate wells for each group). At 30% to 50% confluency, cells were transfected with no plasmid/nanoparticle (Control group), 20 μg blank BSA-NPs to a final concentration of 2 mg/mL (BSA-NPs group), 20 μg of empty pcDNA3.1 using the lipid transfection reagent (Empty vector group), 20 μg of pcDNA3.1-hMDA-7 using the lipid transfection reagent (hMDA-7 plasmid group), and 20 μg of BSA- hMDA-7-NPs to a final concentration of 2 mg/mL (hMDA-7 BSA-NPs group).

### Cell proliferation assessment

Proliferation of BXPC-3 and PANC-1 cells was assessed using the 3-(4,5-Dimethylthiazol-2-yl)-2,5-Diphenyltetrazolium Bromide (MTT) reagent (Invitrogen, USA) according to the manufacturer’s instructions. Briefly, cells were cultured for 24, 48, 72, and 96 hours, respectively; then, 100 μg MTT was added into each well for four additional hours of culture. After careful removal of cell culture media, 150 μL DMSO was added to dissolve the formazan crystals. Finally, OD values were obtained at 570 nm on a microplate reader.

### Cell apoptosis assessment

Cell apoptosis was assessed by the Annexin V/FITC (Takara Bio Company, Japan) method. Briefly, binding buffer, Annexin V/FITC, and PI were added sequentially according to the manufacturer’s instructions, and incubated at room temperature for 5 to 15 minutes in the dark. Apoptosis rates of each group were measured by flow-cytometry. The experiment was repeated three times and average values were obtained.

### Animals

BALB/c nude mice (male, 6 weeks old, 20–25 g) were purchased from the animal facility of Chinese Academy of Sciences (Shanghai, China), and housed in the SPF grade center of the animal facility in the hospital affiliated to Qingdao University. The nude mice were housed in standard plastic boxes paved with wood shavings, and the boxes were replaced twice every week. The sterile purified water and cobalt-60 irradiation-sterilized feed for the nude mice were provided by the Animal Experimental Center, and the nude mice feed food containing a certain percentage of protein, fat, cellulose and various kinds of minerals. The mental state, activity, appetite, behavior, and response to treatment of the experimental animals were observed daily. When the animals became severely ill at anytime prior to the experimental endpoint, regular abdominal drainage and improved feed formula and feeding environment were given. For the animals with significant reduced feeding, marasmus, weight loss, massive ascites and systemic failure, euthanasia was conducted on these experimental animals to alleviate the pain using cervical dislocation. All experiments were in accordance with the guidelines defined by the hospital affiliated to Qingdao University, and the study was approved by the Animal Care and ethics committee of Qingdao University.

### Pancreatic cancer mouse model establishment

Animals were randomly divided into five groups (n = 25 in each group), including Control, BSA-NPs, Empty vector, hMDA-7 plasmid, and hMDA-7 BSA-NPs groups, as defined above.

BXPC-3 or PANC-1 pancreatic cancer cells (1×10^7^) were injected into the subcutaneous tissue of the axilla of nude mice. Tumor volumes reached 0.3 to 0.5 cm^3^ in three weeks. Mice received 5 daily injections of 1.5 μg plasmid DNA, nanoparticles carrying 1.5 μg plasmid DNA, or PBS with same volume, according to the respective groups. Tumor growth was monitored and volumes calculated by the formula: volume (mm^3^) = a×b^2^/2 (a and b were the maximum and minimum tumor diameters, respectively) until week six. When the mice were euthanized the tumors were removed and analyzed by histology.

### hMDA-7 protein expression in pancreatic tissues

Fresh pancreatic tumor tissues were obtained and weighted, and cut into small pieces. Protein extraction was carried out by adding pre-chilled protein extraction reagents containing protease inhibitors. After low speed centrifugation, the supernatant was obtained and subjected to SDS-PAGE. The proteins were finally transferred onto nitrocellulose membranes and blocked with a buffer containing 3% skimmed milk. Mouse anti-human hMDA-7 polyclonal antibodies (Santa Cruz, USA) were used as primary antibodies at 1:2000 dilution for overnight incubation a 4°C; secondary antibodies (Invitrogen, USA) were then added for two hours at room temperature. Membranes were developed and images scanned for subsequent analysis.

### hMDA-7 and VEGF protein expression in pancreatic tumor tissues

Fresh pancreatic tissues were fixed with 10% formaldehyde, followed by paraffin embedding, specimen slicing, and incubation at 4°C overnight. hMDA-7 and VEGF expression levels were assessed by specific immunohistochemical kits (Sigma-Aldrich, St. Louis, MO); cells stained brown were considered to be positive. Twenty high power fields were observed for each slide by microscopy (200×, AFX-II inverted microscope, Nikon, Japan), and average numbers of positive cells were used for statistical analysis.

### Statistical analysis

The SPSS 13.0 software was utilized for statistical analyses; data are mean±standard deviation (SD) and were analyzed by one-way analysis of variance (ANOVA). Inter-group comparison was carried out by *t* test; post hoc analysis of group pairs was performed by the SNK test. *P*<0.05 was considered statistically significant.

## Results

### Nanoparticle morphology

**Fig 1:**
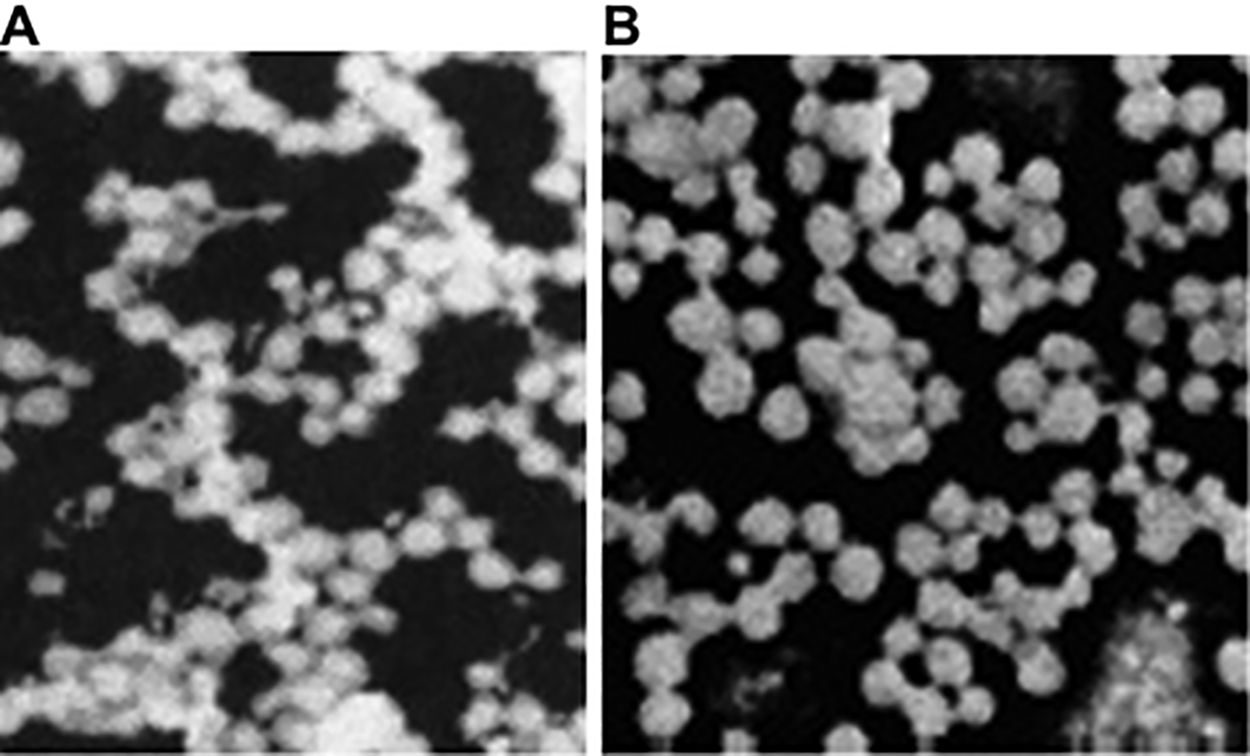
BSA nanoparticle morphology. The nanoparticles were observed by electron microscopy (×20000). A, Empty BSA-NP; B, BSA nanoparticles carrying the hMDA-7 gene.

Nanoparticles were assessed by electron microscopy (Fig 1). Empty BSA-NP and BSA nanoparticles carrying the hMDA-7 gene are displayed in Fig 1A and 1B, respectively. Particle sizes were 115.6±12.3 nm in both groups. The nanoparticles were uniform and spherical. Delivering DNA into cells is challenging due to its negative charge that leads to repulsion by the negative cell membrane. When the DNA is encapsulated within the nanoparticles, the positive charge on the surface of nanoparticles can attract the negative charge of the cell membrane and then make the DNA effectively enter the cell. In our study, the nanoparticles encapsulating the hMDA-7 gene showed the zeta potential values of +33.84+/-4.1 mv, which is favorable for the endocytosis of hMDA-7 gene.

### Treatment with hMDA-7 BSA-NPs results in inhibited BXPC3 and PANC-1 cell proliferation

**Fig 2:**
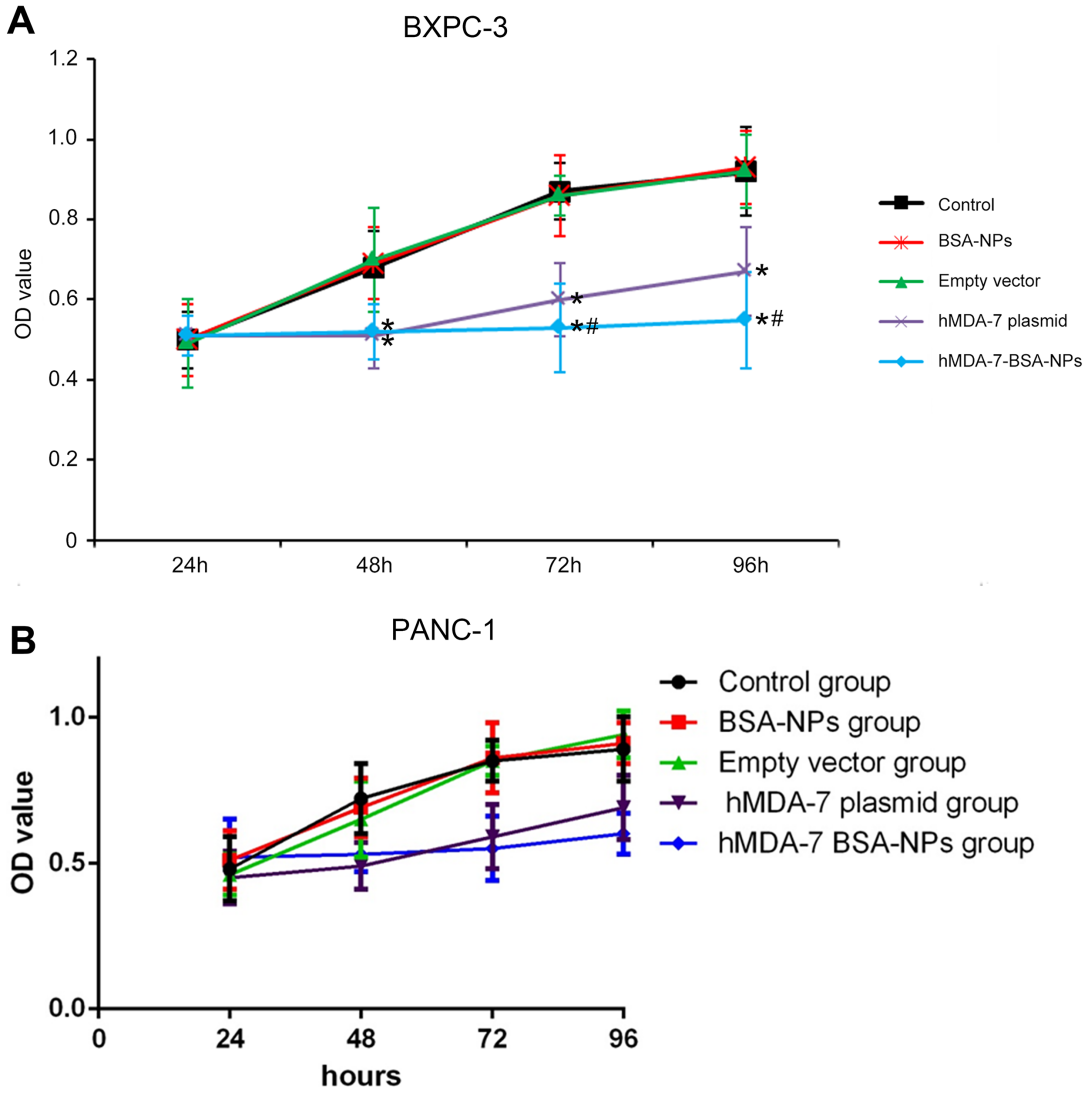
hMDA-7 BSA-NPs inhibit BXPC3 and PANC-1 cell proliferation. BXPC-3(A) and PANC-1 cells(B), respectively, were transfected with no plasmid/nanoparticle (Control group), 20 μg blank BSA-NPs to a final concentration of 2 mg/mL (BSA-NPs group), 20 μg of empty pcDNA3.1 using the lipid transfection reagent (Empty vector group), 20 μg of pcDNA3.1-hMDA-7 using the lipid transfection reagent (hMDA-7 plasmid group), and 20 μg of BSA- hMDA-7-NPs to a final concentration of 2 mg/mL (hMDA-7 BSA-NPs group), and assessed for proliferation by MTT assay. *p<0.05 compared to Control, BSA-NP, and Empty vector groups; #p<0.05 compared to the hMDA-7 plasmid group. All experiments were repeated three times. Data are mean±SD.

Cell proliferation was assessed for various cell groups. As shown in Fig 2, the Control, BSA-NPs, and Empty vector groups showed similar cell growth rates. Meanwhile, the hMDA-7 plasmid and hMDA-7 BSA-NPs groups showed markedly reduced cell viability compared with the above groups, at 48-96h, for both BXPC3 and PANC-1 cells. Importantly, the hMDA-7 BSA-NPs group showed significantly reduced cell viability compared with the hMDA-7 plasmid groups at 72 and 96 h time points for BXPC-3 line (P < 0.05) (Fig 2A). However, no obvious differences was shown between the hMDA-7 plasmid group and the hMDA-7 BSA-NPs group at any time point for the PANC-1 cell line(Fig 2B).

### Treatment with hMDA-7 BSA-NPs results in increased BXPC3 and PANC-1 cell apoptosis

**Fig 3:**
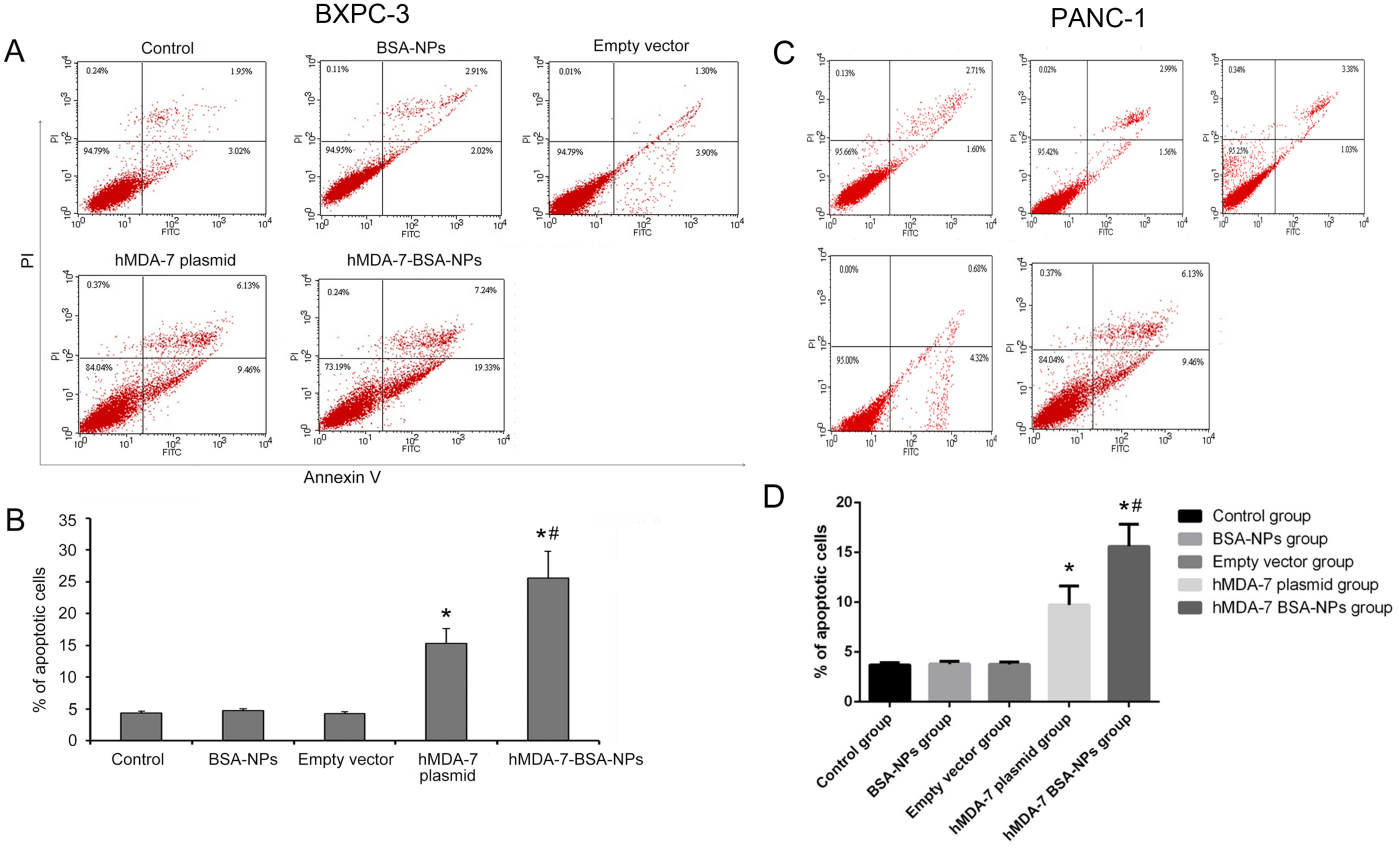
hMDA-7 BSA-NPs enhance BXPC-3 and PANC-1 apoptosis. BXPC-3 and PANC-1 cells, respectively, were transfected with no plasmid/nanoparticle (Control group), 20 μg blank BSA-NPs to a final concentration of 2 mg/mL (BSA-NPs group), 20 μg of empty pcDNA3.1 using the lipid transfection reagent (Empty vector group), 20 μg of pcDNA3.1-hMDA-7 using the lipid transfection reagent (hMDA-7 plasmid group), and 20 μg of BSA- hMDA-7-NPs to a final concentration of 2 mg/mL (hMDA-7 BSA-NPs group), and assessed for apoptosis by flow-cytometry. A and C, flow-cytograms of BXPC-3 and PANC-1 cells, respectively; B and D, quantitation of A and C, respectively. *p<0.05 compared to Control, BSA-NP, and Empty vector groups; #p<0.05 compared to the hMDA-7 plasmid group. All experiments were repeated three times. Data are mean±SD.

Apoptosis rates in BXPC-3 cells were 4.35±0.0.29, 4.71±0.35, 4.28±0.32, 15.30±2.35, and 25.57±4.25% for the Control, BSA-NPs, Empty vector, hMDA-7 plasmid, and hMDA-7 BSA-NPs groups, respectively at 48 hours after transfection. Significant differences were obtained when comparing the hMDA-7 plasmid and hMDA-7 BSA-NPs groups with the Control, BSA-NPs, and Empty vector groups (*P* < 0.05); meanwhile, the hMDA-7 BSA-NPs group showed significantly higher apoptosis rate compared with the other 4 groups (*P* < 0.05) (Fig 3A and 3B). Similar results were obtained with the PANC-1 cell line (Fig 3C and 3D).

### General condition of nude mice

Before the end of the experiments, a total of 3 experimental animals were dead. At the fourth week, 2 nude mice were dead in the BSA-NPs group due to massive ascites and systemic failure. At the fifth week, 1 nude mouse in the empty vector group initially appeared drowsiness, followed by gradually reduced daily feeding, and eventually died of systemic failure. The rest of the experimental animals were alive to the end of the experiment, which included 25 in hMDA-7 plasmid, 25 hMDA-7 BSA-NPs group, 25 in the Control group, 23 in BSA-NPs group, and 24 in Empty vector) groups.

### The hMDA-7 BSA-NPs inhibit tumor growth in vivo

**Fig 4:**
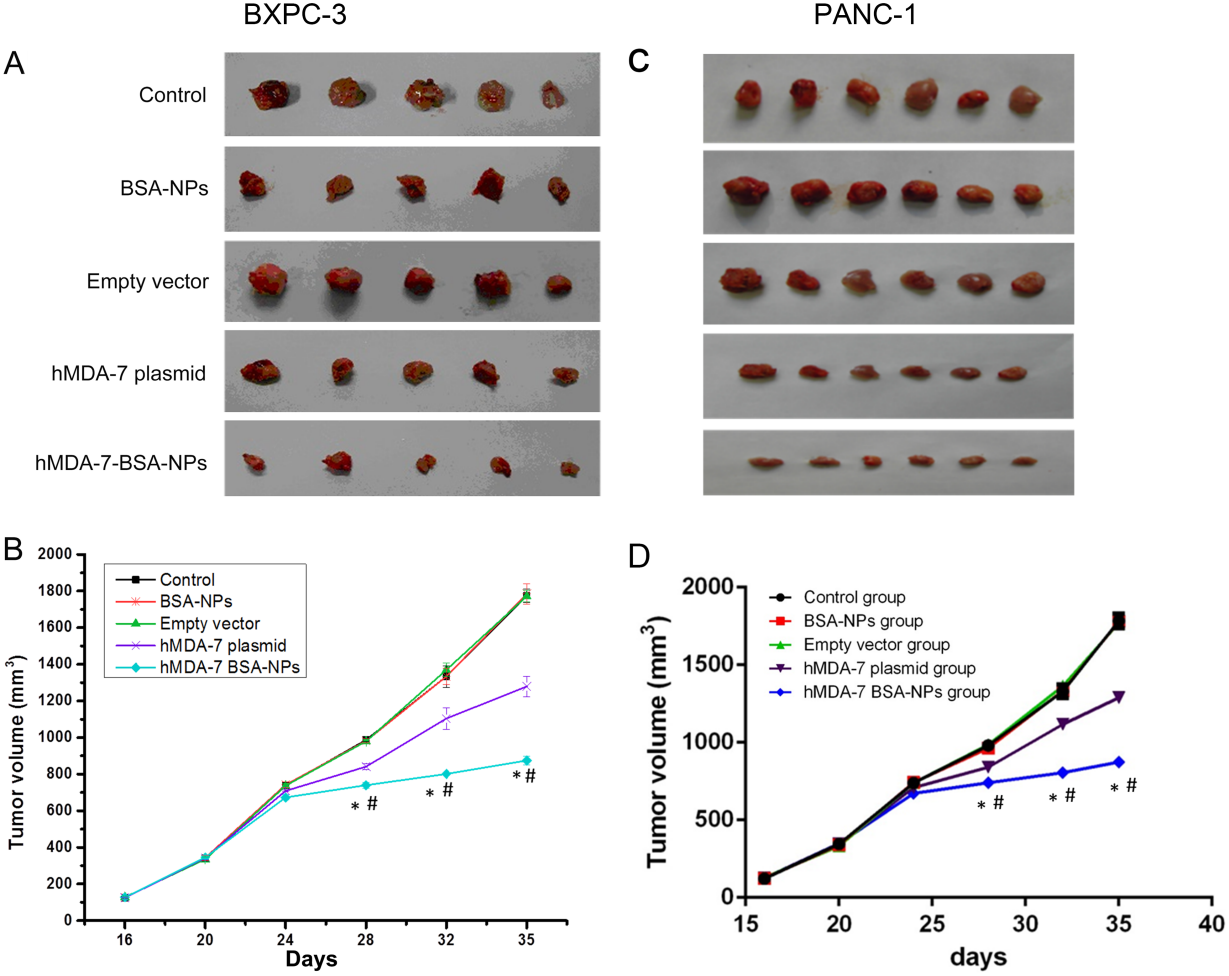
hMDA-7 BSA-NPs decrease pancreatic tumor volumes in BXPC-3 and PANC-1 xenografts. BXPC-3 and PANC-1 cells, respectively, were transfected with no plasmid/nanoparticle (Control group), 20 μg blank BSA-NPs to a final concentration of 2 mg/mL (BSA-NPs group), 20 μg of empty pcDNA3.1 using the lipid transfection reagent (Empty vector group), 20 μg of pcDNA3.1-hMDA-7 using the lipid transfection reagent (hMDA-7 plasmid group), and 20 μg of BSA- hMDA-7-NPs to a final concentration of 2 mg/mL (hMDA-7 BSA-NPs group), and subcutaneously injected into nude mice. A and C, photographs depicting the extracted tumors from BXPC-3 and PANC-1 xenograft models, respectively; B and D, tumor volume quantitation of A and C, respectively. *p<0.05 compared to Control, BSA-NP, and Empty vector groups; #p<0.05 compared to the hMDA-7 plasmid group. All experiments were repeated three times. Data are mean±SD.

Tumors of about 5 mm diameter were observed in nude mice seven days after injection of tumor cells (BXPC-3 and PANC-1 cells), a tumorigenesis rate of 100%. Tumor volumes were progressively enlarged throughout the study. However, significantly slower growth was observed in the hMDA-7 plasmid and hMDA-7 BSA-NPs groups in comparison with the three control (Control, BSA-NPs, and Empty vector) groups. Tumor volumes obtained with BXPC-3 cells were 1786.36±24.20, 1821.30±22.35, 1792.71±20.53, 1329.25±19.25, and 896.24±17.25 mm^3^ for the Control, BSA-NPs, Empty vector, hMDA-7 plasmid, and hMDA-7 BSA-NPs groups, respectively, at 5 weeks. Interestingly, the hMDA-7 plasmid and hMDA-7 BSA-NPs groups showed statistically significant differences compared with the three control groups (*P*<0.05); meanwhile, the hMDA-7 BSA-NPs group also showed significantly differences in tumor volumes compared with the hMDA-7 plasmid group, at this time point (*P* < 0.05) (Fig 4A and 4B). Similar results were obtained in mice injected the PANC-1 cell line (Fig 4C and 4D).

### The hMDA-7 BSA-NPs increase hMDA-7 and VEGF protein expression levels in pancreatic tumor tissues

**Fig 5:**
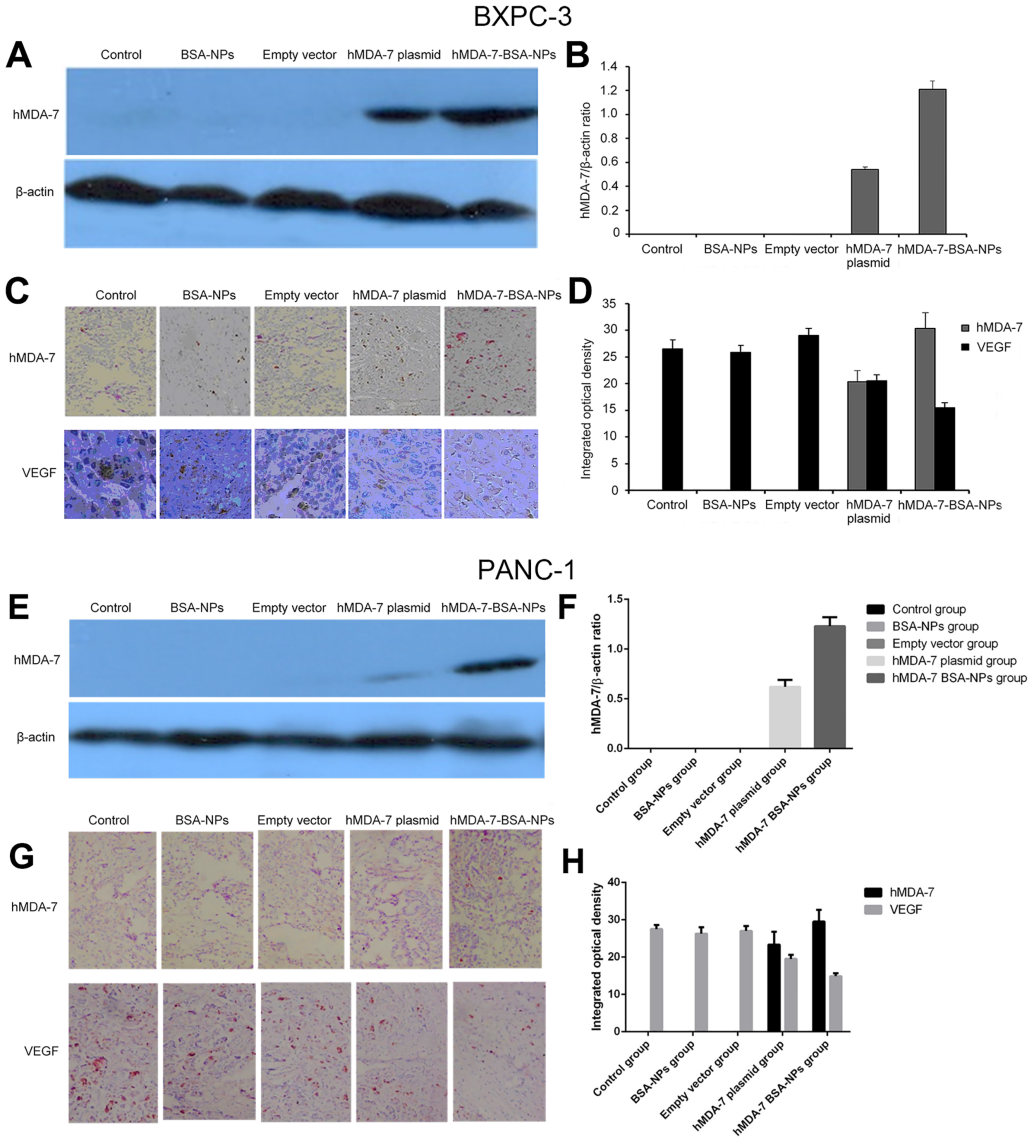
Treatment with hMDA-7 BSA-NPs induces hMDA-7 and represses VEGF expressions in pancreatic cancer BXPC-3 and PANC-1 xenograft tumors. A and E, Western blot membranes showing hMDA-7 and β-actin (used as a loading control) in tumor tissues of the Control, BSA-NPs, Empty vector, hMDA-7 plasmid, and hMDA-7 BSA-NPs groups of BXPC-3 and PANC-1 xenograft models, respectively. B and F, quantitation of A and E, respectively. C and G, immunohistochemical staining for hMDA-7 and VEGF (×200) in tumor tissues of the Control, BSA-NPs, Empty vector, hMDA-7 plasmid, and hMDA-7 BSA-NPs groups of BXPC-3 and PANC-1 xenograft models, respectively. D and H, quantitation of C and G, respectively. All experiments were repeated three times. Data are mean±SD.

Tumors generated by BXPC-3 and PANC-1 cells were removed at week 6 after mouse euthanasia. First, hMDA-7 protein levels in tumor tissues were evaluated by Western blot: no hMDA-7 protein expression was observed in the Control, BSA-NPs, and Empty vector groups; meanwhile, the hMDA-7 protein was detected in the hMDA-7 plasmid and hMDA-7 BSA-NPs groups, with the hMDA-7 BSA-NPs showing significantly higher amounts compared with the hMDA-7 plasmid group (*P*<0.05) in the BXPC-3 (Fig 5A and 5B) and PANC-1 (Fig 5E and 5F) groups. Similar results were obtained by immunohistochemical staining; hMDA-7 positive granules were mainly located in the cell membrane or cytoplasm, as yellow brown color in the BXPC-3 (Fig 5C and 5D) and PANC-1 (Fig 5G and 5H) groups.

In addition, VEGF expression was assessed by immunohistochemistry: VEGF positive (yellow brown) granules were mainly found in the cell membrane or cytoplasm. As shown in Fig 5C and 5D (BXPC-3 xenografts) and Fig 5G and 5H (PANC-1 xenografts), VEGF levels were significantly lower in the hMDA-7 plasmid and hMDA-7 BSA-NPs groups compared with the three respective control groups; the reduction was more pronounced in the hMDA-7 BSA-NPs group compared with the hMDA-7 plasmid group (Fig 5C and 5D).

## Discussion

In this study, we demonstrated that BSA-NP-hMDA-7 effectively decreased BXPC-3 and PANC-1 pancreatic cancer cell proliferation, and increased apoptosis *in vitro*, reducing tumor size and VEGF expression in mouse pancreatic tissues.

As shown above, the BSA nanocarriers were spherical, with good dispersion and uniformed sizes. Their sizes of 115.6±12.3 nm were only slightly increased to 120.7±11.2 nm after 10 days of incubation at 25°C, indicating sufficient stability for *in vitro* and animal studies [15]. Gene therapy, a promising tool for treating pancreatic cancer [9], can be facilitated by the use of nanoparticles for delivery.

Human MDA-7 has broad spectrum anti-tumor activity, selectively inhibiting the proliferation of multiple tumor cell types from diversified tissues; in addition, hMDA-7 has significant anti-angiogenetic effects on liver, colon, and lung cancers, without affecting healthy cells or tissues [16–20]. These findings suggest that hMDA-7 could be used as a novel gene therapeutic for tumor treatment; however, this has not been described for pancreatic cancer. We found that BSA-NP-hMDA-7 effectively transfected pancreatic cancer cells, significantly inhibiting their proliferation and increasing apoptosis, suggesting the usefulness of hMDA-7 in pancreatic cancer treatment.

Interestingly, tumor growth was also inhibited *in vivo* after treatment with hMDA-7 BSA-NPs. Tumor growth is dependent on neovascularization, and pancreatic cancer growth closely associated with the proliferation and differentiation of vascular endothelial cells [21–23]. VEGF is known to promote neovascularization by stimulating vascular endothelial cell differentiation; therefore, overexpression of VEGF during tumorigenesis or cancer progression is highly regarded [24]. Interestingly, hMDA-7 was shown to suppress tumor cell invasion and migration by down regulating MMP-2, MMP-9, and VEGF expression [16], although the detailed mechanisms remain unclear. As shown above, hMDA-7 was highly expressed in tumor tissues transfected with BSA-NP-hMDA-7, while VEGF amounts were significantly reduced compared with the other groups (*P*<0.05). These results indicated that hMDA-7 might inhibit tumor vascularization by downregulating VEGF, which could lead to ischemia and hypoxia in tumor cells, eventually inhibiting the growth, invasion and migration of tumor cells [24].

The BSA nanoparticles prepared in the current study have the advantages of high efficiency, sustained release ability, and gene preservation and persistent expression in the transfected cells. Albumin nanoparticles have been widely suggested for gene therapy [25, 26].

Taken together, our findings indicated that BSA-NP-hMDA-7 effectively transfects pancreatic cancer cells, producing a biologically active hMDA-7 protein.

We used a mouse xenograft design, which does not entirely recapitulate the situation in humans. This is a limitation of the present study. Therefore, more in-depth studies are warranted to confirm our findings.

## Conclusion

In this study, BSA nanoparticles carrying the hMDA-7 gene were successfully generated and effectively transfected BXPC-3 and PANC-1 pancreatic cancer cells, causing decreased malignancy *in vitro* and *in vivo*. In mouse xenografts, treatment with BSA-NP-hMDA-7 resulted in decreased VEGF expression in tumor tissues, suggesting that BSA-NP-hMDA-7 might exert anticancer effects via VEGF suppression in this model. These findings provide a strong basis for further assessment of BSA-NP-hMDA-7 for pancreatic cancer treatment.

## Supporting information

S1 File ARRIVE Guidelines checklist. (DOCX)
