# Supplementary material for: Biological nanoparticles carrying the Hmda-7 gene are effective in inhibiting pancreatic cancer in vitro and in vivo
Source: PLoS One. 2017 Oct 6;12(10):e0185507. doi: 10.1371/journal.pone.0185507 (PMC5630125; doi:10.1371/journal.pone.0185507)

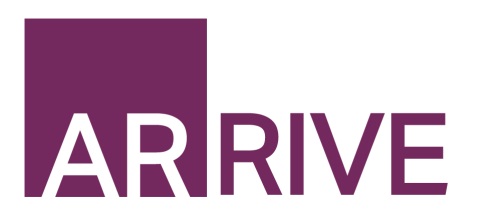


The ARRIVE Guidelines Checklist

Animal Research: Reporting In Vivo Experiments

Carol Kilkenny^1^, William J Browne^2^, Innes C Cuthill^3^, Michael Emerson^4^ and Douglas G Altman^5^

*^1^The National Centre for the Replacement, Refinement and Reduction of Animals in Research, London, UK, ^2^School of Veterinary Science, University of Bristol, Bristol, UK, ^3^School of Biological Sciences, University of Bristol, Bristol, UK, ^4^National Heart and Lung Institute, Imperial College London, UK, ^5^Centre for Statistics in Medicine, University of Oxford, Oxford, UK.*

|  | | ITEM | RECOMMENDATION | Section/ Paragraph |
| --- | --- | --- | --- | --- |
| 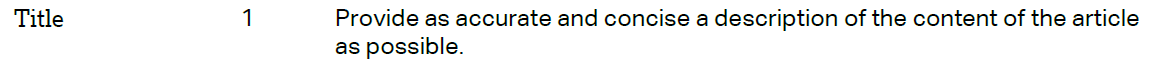 | | | Title, Paragraph 1 |  |
| 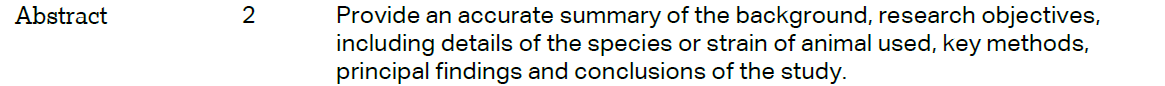 | | | Abstract, paragraph 1 to paragraph 4 |  |
| INTRODUCTION | | |  |  |
| 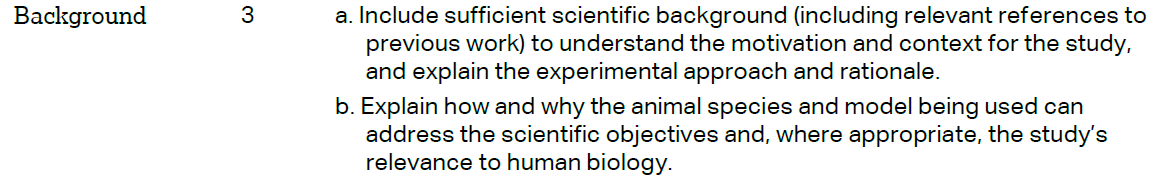 | | | Introduction, paragraph 1 to paragraph 3 |  |
| 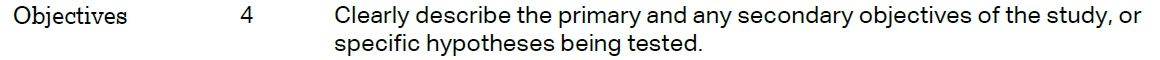 | | | Introduction, paragraph 4 |  |
| METHODS | | |  |  |
| 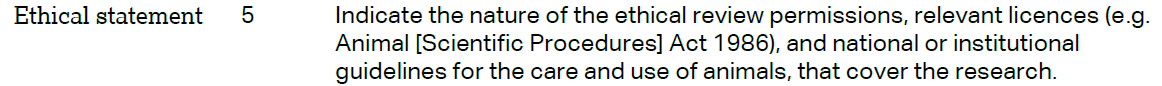 | | | Material and Methods, paragraph 7 |  |
| 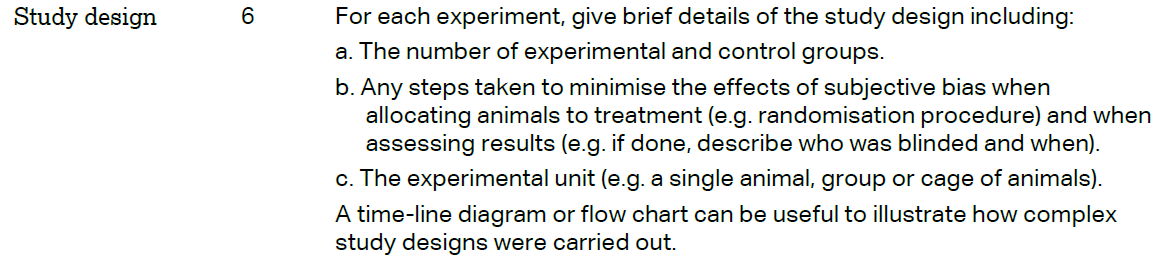 | | | Material and Methods, paragraph 7 to  paragraph 9 |  |
| 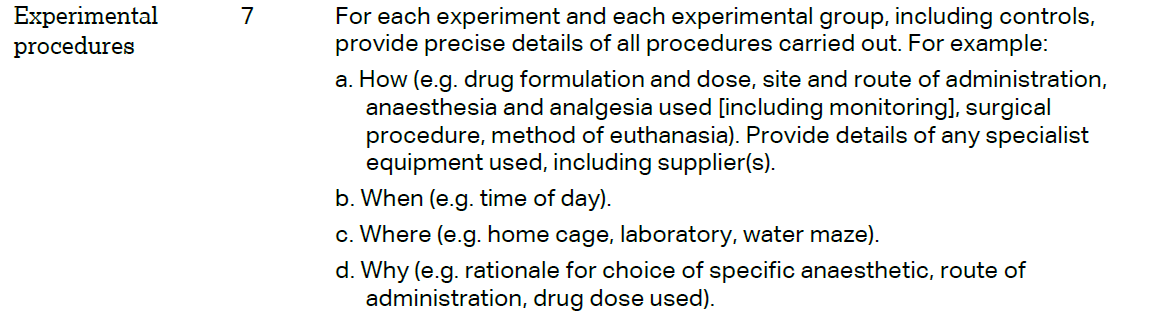 | | | Material and Methods, paragraph 7 to  paragraph 9 |  |
| 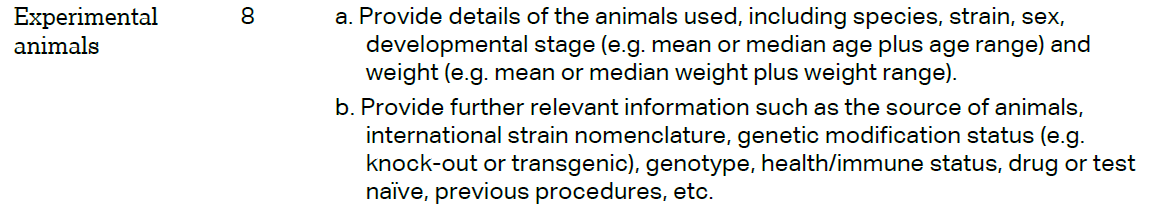 | | | Material and Methods, paragraph 7 to  paragraph 9 |  |

The ARRIVE guidelines. Originally published in *PLoS Biology*, June 2010^1^

| 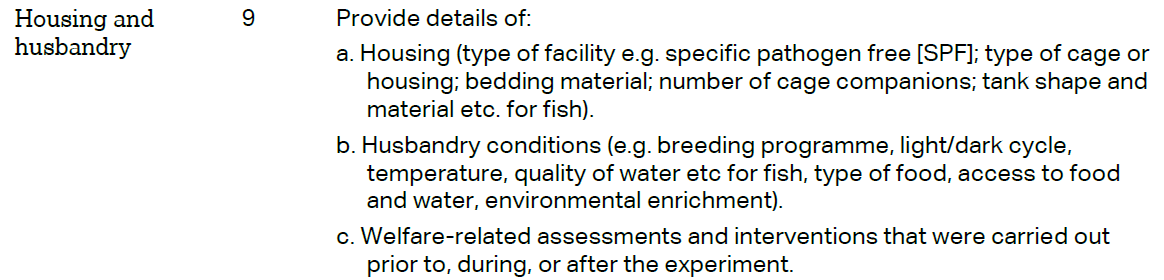 | Material and Methods, paragraph 7 |  |  |
| --- | --- | --- | --- |
| 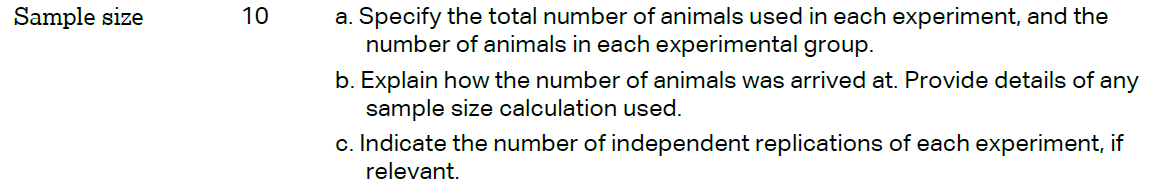 | Material and Methods, paragraph 8 |  |  |
| 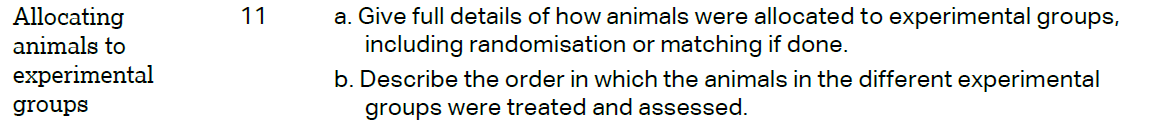 | Material and Methods, paragraph 8 to  paragraph 9 |  |  |
| 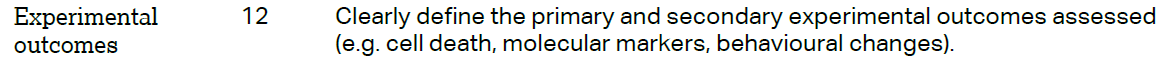 | Material and Methods, paragraph 9 to  paragraph 11 |  |  |
| 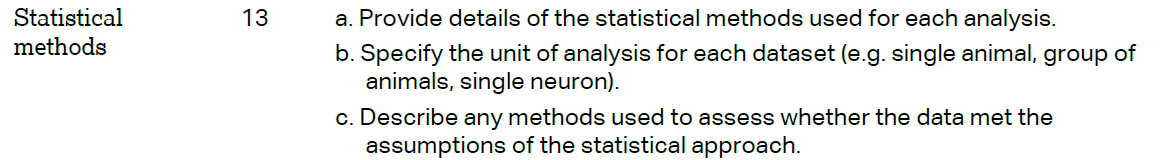 | Material and Methods, paragraph 12 |  |  |
| RESULTS |  |  |  |
| 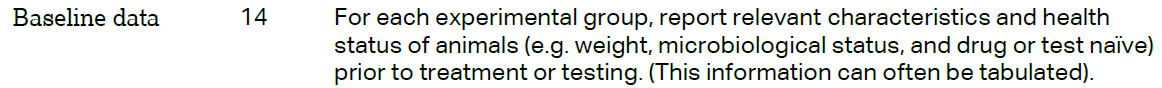 | Results, paragraph 4 |  |  |
| 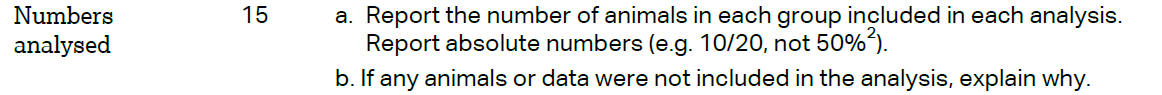 | Results, paragraph 4 |  |  |
| 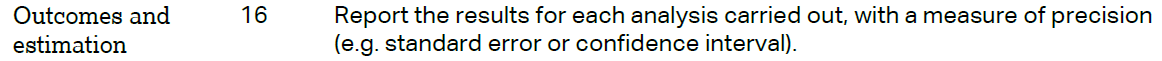 | Results, paragraph 4 |  |  |
| 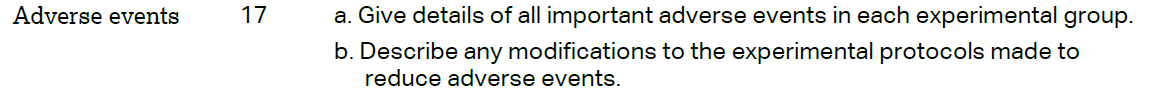 | Results, paragraph 4 |  |  |
| DISCUSSION |  |  |  |
| 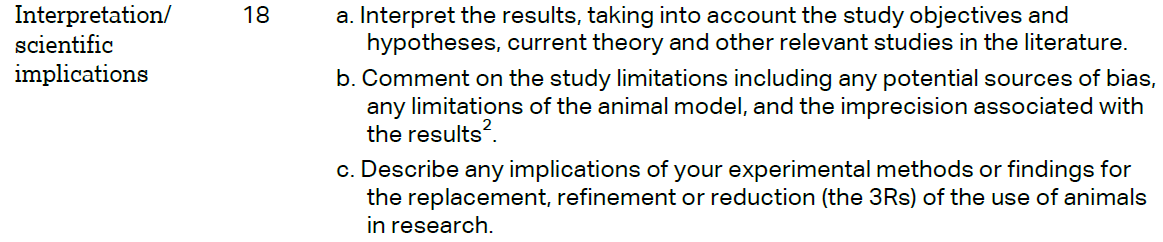 | Discussion, paragraph 1 to paragraph 4 |  |  |
| 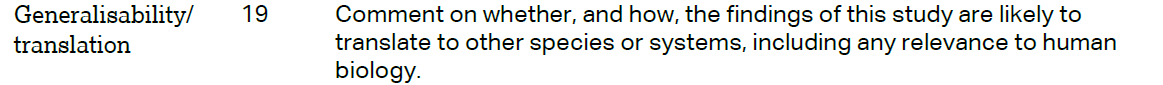 | Discussion,paragraph 4 |  |  |
| 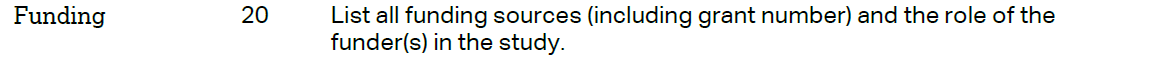 | | Author Contributions, paragraph 1 | |


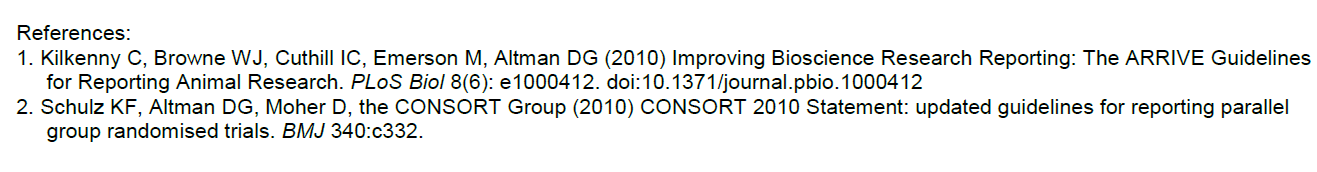

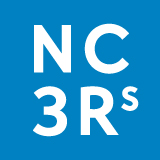

Supplement: S1 File — (DOCX) [file pone.0185507.s001.docx]
